# Co-Culture with Human Osteoblasts and Exposure to Extremely Low Frequency Pulsed Electromagnetic Fields Improve Osteogenic Differentiation of Human Adipose-Derived Mesenchymal Stem Cells

**DOI:** 10.3390/ijms19040994

**Published:** 2018-03-27

**Authors:** Sabrina Ehnert, Martijn van Griensven, Marina Unger, Hanna Scheffler, Karsten Falldorf, Anne-Kristin Fentz, Claudine Seeliger, Steffen Schröter, Andreas K. Nussler, Elizabeth R. Balmayor

**Affiliations:** 1 Siegfried Weller Institute for Trauma Research, Eberhard-Karls-Universität Tübingen, 72076 Tübingen, Germany; sabrina.ehnert@med.uni-tuebingen.de (S.E.); hscheffler@bgu-tuebingen.de (H.S.); sschroeter@bgu-tuebingen.de (S.S.); andreas.nuessler@med.uni-tuebingen.de (A.K.N.); 2 Experimental Trauma Surgery, Klinikum rechts der Isar, Technical University of Munich, 81675 München, Germany; martijn.vangriensven@tum.de (M.v.G.); marina.unger@tum.de (M.U.); claudine.seeliger@tum.de (C.S.); 3 Sachtleben GmbH, 20251 Hamburg, Germany; falldorf@citresearch.de (K.F.); fentz@citresearch.de (A.-K.F.)

**Keywords:** extremely low frequency pulsed electromagnetic fields (ELF-PEMF), primary human osteoblasts (OBs), primary human adipose-derived mesenchymal stem cells (Ad-MSCs), primary human osteoclasts (OCs)

## Abstract

Human adipose-derived mesenchymal stem cells (Ad-MSCs) have been proposed as suitable option for cell-based therapies to support bone regeneration. In the bone environment, Ad-MSCs will receive stimuli from resident cells that may favor their osteogenic differentiation. There is recent evidence that this process can be further improved by extremely low frequency pulsed electromagnetic fields (ELF-PEMFs). Thus, the project aimed at (i) investigating whether co-culture conditions of human osteoblasts (OBs) and Ad-MSCs have an impact on their proliferation and osteogenic differentiation; (ii) whether this effect can be further improved by repetitive exposure to two specific ELF-PEMFs (16 and 26 Hz); (iii) and the effect of these ELF-PEMFs on human osteoclasts (OCs). Osteogenic differentiation was improved by co-culturing OBs and Ad-MSCs when compared to the individual mono-cultures. An OB to Ad-MSC ratio of 3:1 had best effects on total protein content, alkaline phosphatase (AP) activity, and matrix mineralization. Osteogenic differentiation was further improved by both ELF-PEMFs investigated. Interestingly, only repetitive exposure to 26 Hz ELF-PEMF increased Trap5B activity in OCs. Considering this result, a treatment with gradually increasing frequency might be of interest, as the lower frequency (16 Hz) could enhance bone formation, while the higher frequency (26 Hz) could enhance bone remodeling.

## 1. Introduction

Extensive bone loss after trauma or diseases often results in delayed or impaired bone healing [1]. In addition to surgical stabilization, such critical-sized bone defects often require additional intervention to achieve union. Present surgical procedures, such as the Masquelet technique and Ilizarov technique, may reconstruct large bone defects, but require a large volume of bone to fill the defect [2]. The following process of bone regeneration requires recruitment, expansion, and differentiation of mesenchymal stem cells (MSCs) [3], also known as multipotent or mesenchymal stromal cells. Thus, bone tissue engineering with MSCs has great potential to provide the means for inducing osteogenesis and ossification, and thereby consolidation of large bone defects in a timely manner [2]. Up to now, there are over 350 registered clinical trials worldwide, which in different phases, aim at evaluating the cell therapeutic potential of MSCs [4].

MSCs are multipotent cells with a high capacity for self-renewal and the potential to differentiate into several cell types, e.g., bone (osteoblasts), cartilage (chondrocytes), and fat (adipocytes) [5]. The modes of action how MSCs may support bone regeneration are diverse. MSCs may not only differentiate directly into the target cells, but also initiate alternative mechanisms, including secretion of paracrine factors (growth factors, cytokines, and hormones), MSC–cell interactions mediated by tunneling nanotubes, and release of extracellular vesicles or exosomes that contain reparative peptides, mRNA, and microRNAs, may enhance cell viability or proliferation, reduce cell apoptosis, and in some cases, modulate immune responses [6].

MSCs can be isolated from various tissues; most frequently, from bone marrow and adipose tissue. Due to their easy access, low immune rejection, as well as a low risk of tumorigenesis [3,7], MSCs derived from adipose tissue (Ad-MSCs) could be an ideal source for cell-based therapies. However, the osteogenic differentiation potential of these Ad-MSCs has been critically discussed. Both donor age and long-term culture may lower the self-renewal capacity of these cells, resulting in an incomplete differentiation into the committed cell lineage [8,9,10,11]. Transplantation of undifferentiated Ad-MSCs was reported to induce bone formation, however, their osteogenic potential seems to be inferior to bone marrow-derived MSC (B-MSCs) [12]. Overall, the produced bone tissue may not be adequately stiff to serve a load-bearing function, in large bone defects [13].

In the afore-mentioned study, addition of platelet rich plasma could improve the osteogenic differentiation of the Ad-MSCs [12]. Gershovich and colleagues showed enhanced osteogenesis in co-cultures of human B-MSCs with human umbilical vein endothelial cells (HUVECs) [14]. Similarly, Bulnheim and colleagues showed enhanced osteogenesis in co-cultures of human B-MSCs with human dermal microvascular endothelial cells (HDMECs) [15]. Birmingham and colleagues showed that proliferation and osteogenic differentiation of murine B-MSCs is significantly improved when co-cultured with osteoblasts and osteocytes, respectively [16]. These reports accentuate the effect the local bone milieu has on the transplanted cells.

In 1974, Bassett and colleagues reported for the first time that electromagnetic fields might accelerate and improve bone formation [17]. In vivo, electrical and electromagnetic fields are assumed to play a role in bone healing through the same principles as mechanical stress applications. When mechanical load is applied to bone, a strain gradient develops. Resulting pressure gradients in the interstitial fluid cause flow-related shear stress and electrical potentials subsequent to the compensating fluid stream [18]. Both in vitro and in vivo bone cells show increased proliferation as well as production of extracellular matrix and growth factors upon exposure to electromagnetic fields (EMFs) or pulsed electromagnetic fields (PEMFs) [19,20,21]. This is supported by our own work showing that defined extremely low-frequency pulsed electromagnetic fields (ELF-PEMFs) improve the osteogenic differentiation potential of primary human osteoblasts (OBs) [22,23] and human Ad-MSCs [24].

Based on the known literature, we aimed at investigating whether proliferation and osteogenic differentiation potential of Ad-MSCs can be augmented by co-cultures with OBs when compared to the individual mono-cultures. In addition, we aimed to identify the preferred OB/Ad-MSC ratio for osteogenic differentiation in this setting. Moreover, we aimed at analyzing whether the osteogenic differentiation of the co-culture can be further improved by repetitive exposure to two specific ELF-PEMFs identified to improve osteogenic differentiation of OBs and Ad-MSCs in our previous works [22,24], and how these ELF-PEMFs affect primary human osteoclast function (OCs). Functional readout, total protein content, mitochondrial activity, alkaline phosphatase (AP—product of active osteoblast and osteogenic marker [25,26]), and tartrate resistant acidic phosphatase (Trap5B—specifically expressed by osteoclast and osteoclastogenic marker [26]) activity, as well as matrix mineralization (alizarin red staining and von Kossa staining) will be assessed.

## 2. Results

### 2.1. Co-Culture Improves Proliferation of OBs and Ad-MSCs

**Figure 1:**
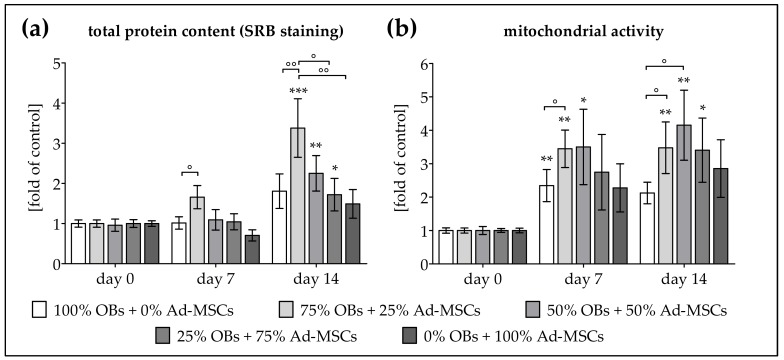
Increased proliferation in co-cultures of human osteoblasts (OBs) and adipose-derived mesenchymal stem cells (Ad-MSCs). OBs and Ad-MSCs (*N* = 6, *n* ≥ 3) were plated as mono- as well as co-cultures with a 3:1, 1:1 or 1:3 ratio and osteogenically differentiated as indicated in the materials and methods section. On days 0, 7 and 14 of differentiation (**a**) total protein content was determined by sulforhodamine B (SRB) staining and (**b**) mitochondrial activity was determined by Resazurin conversion. Results are given as fold of control (average of day 0). * *p* < 0.05, ** *p* < 0.01 and *** *p* < 0.001 as compared to day 0. ° *p* < 0.05 and °° *p* < 0.01 as indicated.

To assess whether OBs and Ad-MSCs profit from co-culture conditions, cells (*N* = 6, *n* ≥ 3) were plated as mono- and co-cultures with OB/Ad-MSC ratios of 3:1, 1:1, and 1:3. The cells’ viability and proliferation were determined by measuring total protein content and mitochondrial activity on days 0, 7, and 14 of osteogenic differentiation. In order to minimize variation due to donor differences, results are given as fold of control, represented by the average of day 0. Total protein content was significantly elevated in the co-cultures. The most prominent effect was seen in the co-culture with 75% OBs + 25% Ad-MSCs, which was ca. 2-fold higher than the respective mono-cultures on day 14 of differentiation (Figure 1a). Similarly, mitochondrial activity was induced by the co-culture condition. On days 7 and 14, mitochondrial activity was strongly induced in the co-cultures with 75% OBs + 25% Ad-MSCs and 50% OBs + 50% Ad-MSCs (~1.5-fold higher than the respective mono-cultures) (Figure 1b).

### 2.2. Co-Culture Improves AP Activity and Matrix Mineralization of OBs and Ad-MSCs

In order to determine whether the co-culture condition also affects the osteogenic function, OBs and Ad-MSCs (*N* = 6, *n* ≥ 3) were plated as mono- as well as co-cultures with OB/Ad-MSC ratios of 3:1, 1:1, and 1:3. The cells’ AP activity and matrix mineralization were determined on days 0, 7, and 14 of osteogenic differentiation. AP was used as marker to assess osteogenesis, due to its clear relevancy in bone formation. AP is a ubiquitous enzyme expressed on the cell membrane of osteoblasts, that plays an important role in osteoid formation and mineralization [25]. This enzyme degrades inhibitors of mineralization at an alkaline pH [26]. AP is the first bone formation marker to be used in both clinical and research settings with wide acceptance and robust results [26].

**Figure 2:**
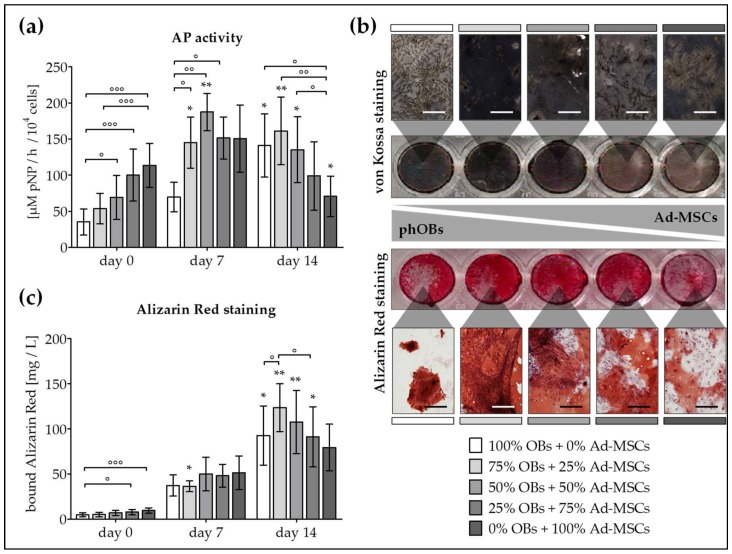
Increased AP activity and matrix mineralization in co-cultures of OBs and Ad-MSCs. OBs and Ad-MSCs (*N* = 6, *n* ≥ 3) were plated as mono- as well as co-cultures with a 3:1, 1:1, or 1:3 OB/Ad-MSC ratio, and osteogenically differentiated as indicated in the Materials and Methods section. On days 0, 7, and 14 of differentiation (**a**) AP activity was measured and (**b**) von Kossa and alizarin red staining was performed to visualize matrix mineralization (100× magnification; representative figure for day 14). (**c**) Matrix mineralization was quantified by resolving the alizarin red staining. * *p* < 0.05 and ** *p* < 0.01 as compared to day 0. ° *p* < 0.05, °° *p* < 0.01 and °°° *p* < 0.001 as indicated. Scale bar = 400 μm.

Basal AP activity was highest in Ad-MSC mono-culture (3.2-fold higher than OB mono-culture). AP activity increased within the first 7 days of differentiation in all settings. During this time, cells profited from the co-culture condition, as highest AP activity was observed in co-cultures with 50% OBs + 50% Ad-MSCs. In co-cultures with more than 50% OBs, AP activity further increased until day 14 (Figure 2a). In line with the AP activity, strongest basal matrix mineralization was observed in Ad-MSC mono-cultures (2-fold higher than OB mono-cultures) as determined by alizarin red staining. Matrix mineralization significantly increased with osteogenic differentiation in all settings. Noteworthy, on day 14 of differentiation strongest von Kossa and alizarin red staining was observed in co-cultures with 75% OBs + 25% Ad-MSCs (1.23 g/L), which represents a 24.2-fold increase compared to day 0 (Figure 2b,c). Based on these data, a co-culture with 75% OBs + 25% Ad-MSCs was chosen for further experiments.

### 2.3. ELF-PEMF Exposure Improves Viability and Function of OB and Ad-MSC Co-Cultures

**Figure 3:**
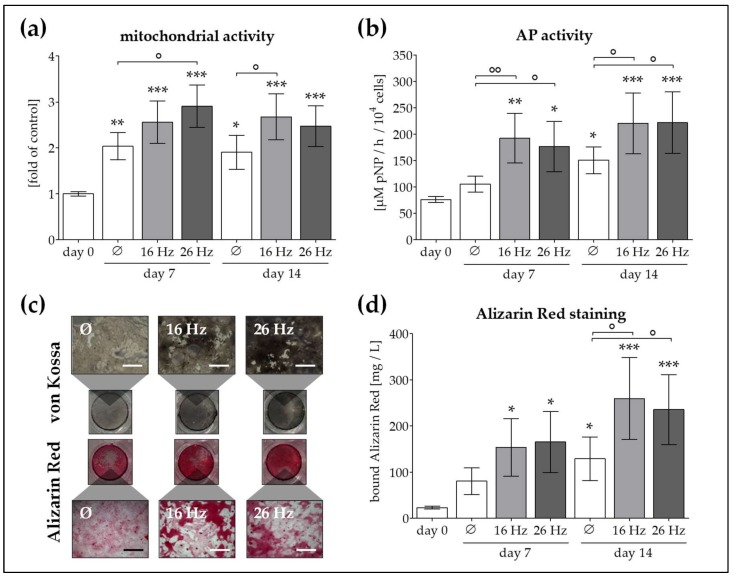
ELF-PEMF application with the Somagen^®^ device improved function of OBs and Ad-MSCs co-culture. OBs and Ad-MSCs (*N* = 6, *n* ≥ 3) were plated as co-culture with a 3:1 ratio. During the osteogenic differentiation process, cells were exposed to two specific ELF-PEMFs (16 and 26 Hz) 5 times per week, for 7 min each day. ELF-PEMFs were generated using the Somagen^®^ device. On days 0, 7, and 14 of differentiation (**a**) mitochondrial activity (resazurin conversion) and (**b**) AP activity were measured. (**c**) von Kossa and alizarin red staining were performed to visualize matrix mineralization (100× magnification). (**d**) Matrix mineralization was quantified by resolving the alizarin red staining. * *p* < 0.05, ** *p* < 0.01 and *** *p* < 0.001 as compared to day 0. ° *p* < 0.05 and °° *p* < 0.01 as indicated. Scale bar = 400 μm.

In the next step, we attempted to investigate whether cell viability and osteogenic function can be further improved by ELF-PEMF exposure. During the entire differentiation process, co-cultures (75% OBs + 25% Ad-MSCs, *N* = 6, *n* ≥ 3) were exposed to ELF-PEMFs 5 times per week, for 7 min each day. We compared the effect of 2 specific ELF-PEMFs (16 and 26 Hz), that proved to be most effective in OB and Ad-MSC mono-cultures [22,24]. After 0, 7 and 14 days of differentiation in presence or absence of ELF-PEMF exposure, mitochondrial activity, AP activity, and matrix mineralization were determined. Mitochondrial activity was increased by ELF-PEMF exposure (1.3 to 1.5-fold as compared to the corresponding unexposed cells) both on day 7 and 14 of differentiation (Figure 3a). AP activity was significantly increased by ELF-PEMF exposure (1.5- to 1.8-fold compared to the corresponding unexposed cells) throughout the entire differentiation process (Figure 3b). The observed early increase in AP activity results in increased matrix mineralization both on day 7 and on day 14 of differentiation in ELF-PEMF treated cells, as determined by von Kossa and alizarin red staining (Figure 3c,d). Interestingly, no significant difference between the two specific ELF-PEMFs was observed.

### 2.4. Increased OC Function by Repetitive Exposure to 26 Hz ELF-PEMF but Not 16 Hz ELF-PEMF

After observing the positive effects of both ELF-PEMFs (16 and 26 Hz) on viability and osteogenic function in our co-culture (75% OBs + 25% Ad-MSCs), we further compared the effect of these ELF-PEMFs on OCs. Hence, we generated OCs from human peripheral blood mononuclear cells (*N* = 6, *n* = 3) in the presence of recombinant human M-CSF and RANKL, as indicated in Materials and Methods. In this case, Trap5B was selected as marker to assess OC function. Trap5B is specifically expressed by osteoclasts and it cleaves type 1 collagen into fragments during bone resorption [26]. Trap5B is widely used in research as bone resorption and osteoclast marker, and it is typically increased in high bone turnover conditions [27].

**Figure 4:**
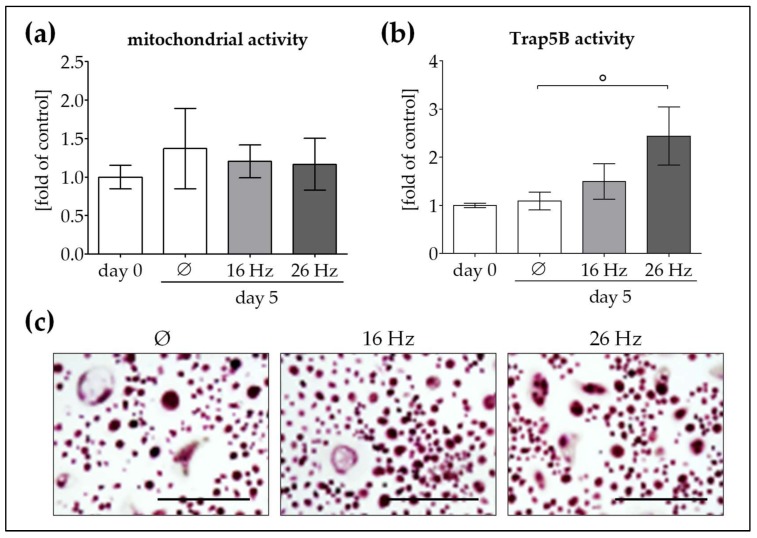
Osteoclast (OC) function is induced by repetitive exposure to 26 Hz ELF-PEMF but not 16 Hz ELF-PEMF. To investigate the effect of the two specific ELF-PEMFs (16 and 26 Hz) on OC function, we differentiated human peripheral blood mononuclear cells (*N* = 6, *n* = 3) to OCs. Following differentiation, OCs were exposed to the two ELF-PEMFs for 7 min each day. TRAP staining and activity was performed, not only to confirm the phenotype of generated OCs, but also to assess the effect of ELF-PEMFs exposure on their function. Thus, after the 5th exposure (day 5) (**a**) mitochondrial activity (resazurin conversion) and (**b**) Trap5B activity were measured. (**c**) Trap5B staining was performed to visualize OCs (100× magnification). ° *p* < 0.05 as indicated. Scale bar = 400 μm.

After 21 days of differentiation, OCs were repetitively exposed to either 16 Hz ELF-PEMF or 26 Hz ELF-PEMF for 7 min each day. After the 5th exposure (day 5), mitochondrial activity and Trap5B activity were measured. In OCs, repetitive ELF-PEMF exposure did not significantly alter mitochondrial activity (Figure 4a). Interestingly, Trap5B activity was not affected when cells were repetitively exposed to 16 Hz ELF-PEMF, but increased (2.2-fold as compared to the corresponding unexposed cells) when cells were repetitively exposed to 26 Hz ELF-PEMF (Figure 4b). It was noteworthy that Trap5B staining revealed no significant changes in morphology between the ELF-PEMF exposed and unexposed OCs (Figure 4c).

## 3. Discussion

In the present project, we investigated whether co-culture of OBs and Ad-MSCs, with different ratios, improves their proliferation and osteogenic differentiation potential compared to the individual mono-cultures. All co-culture conditions showed increased total protein content and mitochondrial activity as compared to the respective mono-cultures, which refers to increased proliferation. This is in line with the work of Heino and colleagues, which showed that conditioned medium from an osteocytic cell line (MLO-Y4), but not an osteoblastic cell line (MC3T3-E1), was able to improve proliferation and osteogenic differentiation of mouse B-MSCs [28], suggesting that the observed effect is triggered by factors released from the osteocytes. Similarly, the work of Birmingham and colleagues showed enhanced cell proliferation of murine B-MSCs when co-cultured with osteoblastic cells [16]. This is in line with our findings, where the co-cultures showed enhanced proliferation when compared to the individual mono-cultures.

In the work of Birmingham and colleagues, however, only co-cultures with osteocytes improved AP activity and matrix mineralization of MSCs [16]. Santos and colleagues even showed inhibitory effects of B-MSCs on osteogenic differentiation of osteoblasts [29]. This is contrary to our findings, where the co-cultures favored AP activity and matrix mineralization, as compared to the individual mono-cultures. Our results are supported by the findings of Glueck and colleagues, who could improve osteogenic differentiation of human B-MSC in a transwell approach with OBs in a 1:1 ratio [30]. In our setting, where OBs and Ad-MSCs are in direct cell–cell contact, a co-culture with 75% OBs and 25% Ad-MSCs showed highest proliferation and osteogenic function. The observed discrepancies with some of the reported studies might be explained by differences in the osteogenic differentiation potential of MSCs isolated from different sources. Ad-MSCs might have a better osteogenic potential than B-MSCs when exposed to certain factors, such as PDGF-BB [29]. Our results presented here indicate that Ad-MSCs also improve their osteogenic potential when co-cultured with osteoblastic cells.

For almost half a century now, electric and electromagnetic fields have been used in vitro and in vivo to accelerate and improve bone formation. However, the effect on bone seems to be strongly dependent on the field parameters chosen [31]. Thus, in the next step, we wanted to investigate whether exposure to specific ELF-PEMFs further improves the osteogenic differentiation potential of our co-culture system. For these experiments, we chose two defined ELF-PEMFs that proved to be most effective in OB and Ad-MSC mono-cultures [22,24]. ELF-PEMFs were generated with the Somagen^®^ device (Sachtleben GmbH, Hamburg, Germany), which is certified for medical use according to European laws. Exposure to either one of the two ELF-PEMFs (16 or 26 Hz) slightly increased proliferation/mitochondrial activity in our co-cultures. This is in line with the observations of Zhou and colleagues showing enhanced protein synthesis and activation of signaling cascades involved in proliferation and cell survival upon exposure to different forms of EMFs (sinusoidal, triangular, square, and serrated) all set at 50 Hz EMFs [20]. As a measure of osteogenic differentiation potential, most frequently, the effect of PEMF on structural extracellular matrix proteins has been explored. Ciombor and colleagues suggest that the application of electric and electromagnetic fields, both in vitro and in vivo, stimulate the synthesis of structural proteins of the extracellular matrix [19]. Also, Zhou et al. showed increased AP activity and matrix mineralization in rat calvarial osteoblasts exposed to sinusoidal EMFs (50 Hz) with an intensity of 1.8 and 3.6 mT [20]. A similar effect is seen in our co-cultures, where ELF-PEMF exposure strongly induced AP activity and matrix mineralization. However, no significant difference was observed between the two different ELF-PEMFs applied, which differ in their frequency by 10 Hz. Our results may be explained by studies, including our own, showing increased expression of transcription factors and growth factors, which enhance cellular repair and the synthesis of extra-cellular matrix proteins [18,22,24]. Zhang and colleagues showed an increased calcium uptake in osteoblasts treated with ELF-PEMF in the range of 50 Hz and 0.8 mT [32].

In our previous work, we could show that improved osteogenic function in ELF-PEMF treated OBs and Ad-MSCs is strongly dependent on MAP kinase activation [22,24], resulting from increased formation of reactive oxygen species, i.e., superoxide and hydrogen peroxide [23]. Depending on the level of reactive oxygen species, also, OCs might be activated [33]. Hong and co-workers [34] showed that EMF-controlled osteoclast activity can be inversely correlated to the osteoblast activity in a murine model. Furthermore, Rubin and colleagues reported that formation of murine osteoclasts might even be suppressed by low frequency, low intensity electric fields [35]. Therefore, in the last step of this project, we wanted to compare how the two specific ELF-PEMS affect OC function. Interestingly, exposure to either of the two ELF-PEMFs (16 or 26 Hz) did not affect mitochondrial activity in OCs. Conversely, as a measure of OC function, Trap5B activity was induced only by 26 Hz ELF-PEMF and not by 16 Hz ELF-PEMF.

Considering a balanced bone formation and bone resorption as crucial for successful bone healing, both explored ELF-PEMFs might be of interest. ELF-PEMF exposure with the Somagen^®^ device applying 16 Hz, was reported to have the strongest effects on OBs mono-cultures [22,23], and equally improved osteogenic function in our co-culture (OBs + Ad-MSCs). Thus, application of 16 Hz ELF-PEMF might be more useful early in bone healing, when large amounts of bone matrix have to be formed. On the other hand, exposure to 26 Hz ELF-PEMF, reported to have the strongest effects on Ad-MSCs mono-cultures [24], comparably improved osteogenic function in our co-culture (OBs + Ad-MSCs). However, OC function was also induced, which could be a sign for higher bone turnover. Thus, increasing the frequency by 10 Hz might be more useful later in bone healing during bone remodeling.

Summarizing our results, co-culture of OBs and Ad-MSCs improves their proliferation and osteogenic differentiation potential when compared to the individual mono-cultures. An OB to Ad-MSC ratio of 3:1 had the strongest increase of the total protein content, mitochondrial activity, AP activity, and matrix mineralization. Furthermore, osteogenic differentiation of this co-culture can be further improved by the two investigated ELF-PEMFs (16 and 26 Hz), which have been shown to be effective in OB and Ad-MSC mono-cultures before [22,23,24]. Interestingly, comparing the effect of the two specific ELF-PEMFs on OC function revealed that only the 26 Hz ELF-PEMF increased Trap5B activity. Considering this result, a combinatory treatment might be of interest, as exposure to 16 Hz ELF-PEMF could enhance bone formation, while exposure to 26 Hz ELF-PEMF could enhance bone remodeling.

## 4. Materials and Methods

rhM-CSF and rhRANKL were purchased from Peprotech (Hamburg, Germany). Chemicals, cell culture medium, and supplements were purchased from Sigma-Aldrich (Munich, Germany).

### 4.1. Ethics Statement

All human studies were performed in accordance with the Declaration of Helsinki in its latest amendment. Immature OBs were isolated from bone tissue explants of patients undergoing total joint replacement. Ad-MSCs were isolated from adipose tissue of patients undergoing reconstructive procedures. OCs were generated from peripheral blood mononuclear cells of the respective patients. Tissues were obtained in accordance with the ethical vote (387/2012BO (7 August 2012) and 385/2012BO (2 August 2012)—Eberhard Karls Universität Tübingen and 5936/13 (5 September 2013)—Klinikum rechts der Isar) and the patients’ written consent. Tissues from (potential) tumor patients or patients with viral or bacterial infections were excluded from this study. The donors’ average age for OB isolation was 72.0 ± 12.9 years [age range: 55–92 years] (3 men and 9 women), and for Ad-MSC isolation was 61.6 ± 11.8 years [age range: 47–82 years] (6 men and 6 women).

### 4.2. Isolation and Expansion of OBs

Briefly, cancellous bone was disintegrated mechanically and washed 3–5 times with Dulbecco’s phosphate buffered saline (DPBS). After 1 h of collagenase digestion (DPBS, 0.07% collagenase II—Biochrom AG, Berlin, Germany) at 37 °C, cancellous bone pieces were washed with DPBS and released OBs were transferred to cell culture flasks in growth medium (MEM/Ham’s F12, 10% FCS, 2 mM l-glutamine, 100 U/mL penicillin, 100 μg/mL streptomycin, 50 μM l-ascorbate-2-phosphate, 50 μM β-glycerol phosphate) for expansion. Medium was changed every 3–4 days [22,23].

### 4.3. Isolation and Expansion of Ad-MSCs

Briefly, adipose tissue was cut into small pieces and washed 3–5 times with DPBS. After 1 h of collagenase digestion (DPBS, 0.07% collagenase II) at 37 °C, remaining connective tissue was removed from the Ad-MSCs using a cell strainer. Ad-MSCs were washed with DPBS and transferred to cell culture flasks in growth medium (DMEM with 4.5 g/L glucose, 10% FCS, 2 mM l-glutamine, 100 U/mL penicillin, 100 μg/mL streptomycin) for expansion. Medium was changed every 3–4 days [36].

### 4.4. Osteogenic Differentiation of OBs and Ad-MSCs Mono- and Co-Cultures

**Figure 5:**
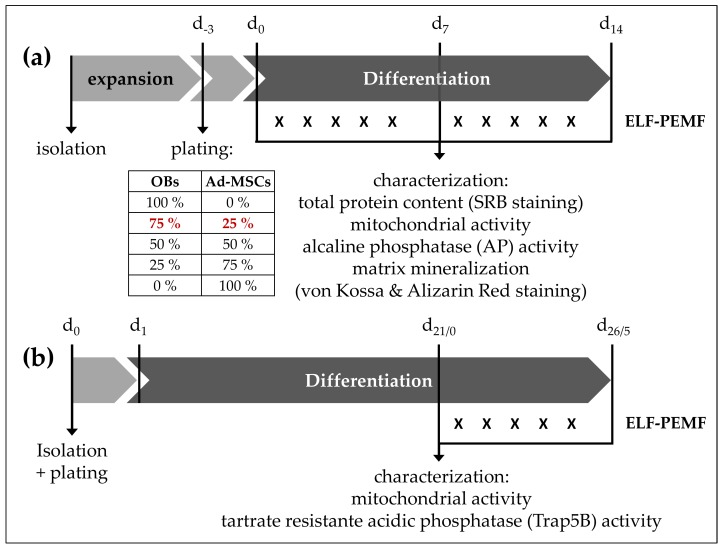
Experimental setup. (**a**) OBs and Ad-MSCs: Cells were plated either as mono- or co-culture with different ratios. After complete adherence (3 days), growth medium was replaced by osteogenic differentiation medium. Medium was replaced every 3–4 days. During the entire differentiation period, cells were exposed daily to ELF-PEMF (16 or 26 Hz) for 7 min (for 5 consecutive days). On day 0, 7, and 14, total protein content (SRB staining), mitochondrial activity (resazurin conversion), AP activity, and matrix mineralization (alizarin red and von Kossa staining) were assessed. The red numbers 75% (OB) and 25% (Ad-MSCs) indicate the co-culture ratio with the best results. (**b**) Generated OCs: Mononuclear cells were isolated from peripheral blood via density gradient centrifugation. After 24 h, non-adherent cells were washed off the plate, and cells were exposed to osteoclastic (de-)differentiation medium, as indicated in Materials and Methods. Medium was replaced every 3–4 days over the course of 21 days. After 21 days (indicated as d21/0), the generated OCs were exposed daily to ELF-PEMF (16 or 26 Hz) for 7 min each day for the following 5 days. Before and after ELF-PEMF exposure, mitochondrial activity and Trap5B activity were measured. “X” indicates the daily 7 min exposure of the cells or cell cultures to ELF-PEMF.

Experiments were performed in passage 3, when pure populations of OBs and Ad-MSCs were reached. Ad-MSCs were tested by flow cytometry, and only used when they were negative for CD14 and CD45, and positive for CD73, CD90, and CD105 [36]. OB phenotype was confirmed by alkaline phosphatase staining and activity determinations, as well as measurements of secreted osteocalcin, as described elsewhere [37]. Osteocalcin expression is restricted to cells of the osteoblastic lineage, and is frequently used as marker for osteoblasts. Cells were seeded at a density of 20,000 cells/cm^2^ in OB growth medium either as mono- or as co-cultures with different ratios (75% OBs + 25% Ad-MSCs, 50% OBs + 50% Ad-MSCs, or 25% OBs + 75% Ad-MSCs). After 3 days, growth medium was replaced by osteogenic differentiation medium (DMEM with 1 g/L glucose, 5% FCS, 2 mM l-glutamine, 100 U/mL penicillin, 100 μg/mL streptomycin, 200 μM l-ascorbate-2-phosphate, 10 mM β-glycerol phosphate, 25 mM HEPES, 1.5 mM CaCl_2_, 100 nM dexamethasone). Medium was changed every 3–4 days for 14 days. For an overview on co-culture and differentiation setup, please see Figure 5a.

### 4.5. Generation of OCs

For OC generation, human mononuclear cells were isolated from whole blood samples by density gradient centrifugation [38] and plated at a density of 3.0 × 10^6^ cells/cm^2^ in isolation medium (α-MEM, 10% FCS, 2 mM l-glutamine, 100 U/mL penicillin, 100 μg/mL streptomycin). Following overnight adherence, medium was changed to de-differentiation medium (α-MEM, 10% FCS, 2 mM l-glutamine, 100 U/mL penicillin, 100 μg/mL streptomycin, 2.5 ng/mL rhM-CSF) for 6 days. OC differentiation was induced by osteoclastic differentiation medium (α-MEM, 10% FCS, 2 mM l-glutamine, 100 U/mL penicillin, 100 μg/mL streptomycin, 20 ng/mL rhRANKL) until day 21. For overview on OC generation setup, please see Figure 5b.

In order to confirm the phenotype of generated osteoclasts, dentine chip assay and TRAP staining were performed as described elsewhere [39,40]. Toluidine blue staining was performed after culturing osteoclasts on dentine chips. Thereby, the formation of resorption lacunae could be visualized. TRAP staining indicated the presence of multinucleated cells as important characteristic of osteoclasts. In addition, TRAP activity was evaluated as an indication of positive osteoclast generation.

### 4.6. Electromagnetic Field Application with the Somagen^®^ Device

Cells were exposed to two specific ELF-PEMFs 5 times per week for 7 min each day. The ELF-PEMFs were generated by the Somagen^®^ (Sachtleben GmbH, Hamburg, Germany), a medical device certified according to European law (CE 0482, compliant with EN ISO 13485:2012 + AC:2012), as previously described [22,23,24]. The specific ELF-PEMFs applied here have an intensity of 6–282 μT (B field amplitude 6 mm above the applicator), which is emitted as groups of pulses (bursts) in sending-pause intervals. The ELF-PEMF with a fundamental frequency of 16 Hz proved to be most effective for OB mono-cultures [22,23]. The ELF-PEMF with a fundamental frequency of 26 Hz proved to be most effective for Ad-MSC mono-cultures [24]. ELF-PEMF exposure was performed for 7 min each day during 5 consecutive days. The stimulation set-up for the co-cultures is depicted in Figure 5a and for OCs in Figure 5b.

### 4.7. Sulforhodamine B (SRB) Staining to Assess Total Protein Content

SRB staining on ethanol fixed cells was performed as reported [41], in order to assess the total protein content. Resolved stain was quantified photometrically (*λ* = 565–690 nm/Omega plate-reader, BMG Labtech, Ortenberg, Germany). Results are given as fold of control (average of day 0). Furthermore, relative cell numbers could be determined by using cell-specific standard curves for the cellular protein content measurements.

### 4.8. Resazurin Conversion Assay to Assess Mitochondrial Activity

For measuring mitochondrial activity, 1/10 volume of a 0.025% (*w*/*v*) resazurin solution (in DPBS) was added to the cells. After 1 h of incubation at 37 °C, fluorescence intensity was measured (ex/em = 540/590 nm/Omega plate-reader) and corrected to background control (solvent mixture without cells). Results are given as fold of control (average of day 0).

### 4.9. AP Activity Measurement

For measuring AP activity, cell cultures (OBs and/or Ad-MSCs) were incubated with reaction buffer (0.2% *p*-nitrophenyl phosphate, 50 mM glycine, 1 mM MgCl_2_, 100 mM TRIS, pH 10.5) at 37 °C. Resulting formation of *p*-nitrophenol (pNP) was determined photometrically (*λ* = 405 nm/Omega plate-reader) and corrected to background control (solvent mixture without cells). Signal intensities were normalized to relative cell numbers by SRB staining.

### 4.10. Assessing Matrix Mineralization by von Kossa and Alizarin Red Staining

For assessing matrix mineralization cell cultures (OBs and/or Ad-MSCs) were fixed with ethanol. After washing 3 times with tap water, cells were incubated with the corresponding staining solutions: (i) for von Kossa staining, 3% silver-nitrate solution for 30 min at ambient temperature, followed by 3 additional washing steps and incubation with sodium carbonate–formaldehyde solution (0.5 M sodium carbonate, 10% formaldehyde) for color development (brownish-black). The resulting staining was assessed microscopically; (ii) for alizarin red staining 0.5% alizarin red solution (pH 4.0) for 30 min at ambient temperature. After 3 additional washing steps, the resulting staining was assessed microscopically. For photometric quantification (*λ* = 562 nm/Omega plate-reader) alizarin red staining was resolved with 10% cetylpyridium chloride solution [22].

### 4.11. Trap5B Activity Measurement

Trap5B activity was measured in culture supernatants of OCs. Culture supernatant (30 μL) was mixed with 90 μL Trap5B activity assay buffer (0.2% *p*-nitrophenyl phosphate, 100 mM Na-acetate, 50 mM Na_2_-tartrate, pH 5.5) and incubated for 1 h at 37 °C. Reaction was stopped by adding 90 μL of 1 M NaOH. Resulting formation of *p*-nitrophenol (pNP) was determined photometrically (*λ* = 405 nm/Omega plate-reader) and corrected to background control (medium without cells). Results are given as fold of control (average of day 0 = day 21 of differentiation).

### 4.12. Trap5B Staining

For Trap5B staining, OCs were fixed with 0.1% Triton X-100 solution (in 3.7% formaldehyde) for 5 min. Then cells were incubated with Trap5B staining solutions (0.01% napthol AS-MX phosphate, 0.06% Fast Red Violet LB salt, 1% *N*-*N*-dimethylformamide, 40 mM Na-acetate, 10 mM Na_2_-tartrate, pH = 5.0) for ca. 15 min at 37 °C. After washing 3 times with DPBS, the resulting staining was assessed microscopically [42].

### 4.13. Statistics

Results are expressed as bar diagrams (mean with 95% CI) of 6 independent experiments (*N* = 6), measured as triplicates or quadruplicates (*n* = 3 or 4). Details are given in the figure legends. Data sets were compared by non-parametric one-way analysis of variance (Kruskal–Wallis) followed by Dunn’s multiple comparison test for each time point or condition individually (GraphPad Prism 6.0 Software, El Camino Real, CA, USA). *p* < 0.05 was taken as minimum level of significance.

## Abbreviations

Ad-MSCs	primary human adipose-derived mesenchymal stem cells
B-MSCs	bone marrow-derived mesenchymal stem cells
AP	alkaline phosphatase
ELF-PEMF	extremely low frequency pulsed electromagnetic fields
FCS	fetal calf serum
hrM-CSF	human recombinant macrophage colony-stimulating factor
hrRANKL	human recombinant receptor activator of nuclear factor κ-B ligand
OBs	primary human osteoblasts
OCs	primary human osteoclasts
Trap5B	tartrate resistant acidic phosphatase
